# Circulating miRNAs Associated with Dysregulated Vascular and Trophoblast Function as Target-Based Diagnostic Biomarkers for Preeclampsia

**DOI:** 10.3390/cells9092003

**Published:** 2020-08-31

**Authors:** Suji Kim, Minsik Park, Ji-Yoon Kim, Taesam Kim, Jong Yun Hwang, Kwon-Soo Ha, Moo-Ho Won, Sungwoo Ryoo, Young-Guen Kwon, Young-Myeong Kim

**Affiliations:** 1 Departments of Molecular and Cellular Biochemistry, School of Medicine, Kangwon National University, Chuncheon, Gangwon-do 24341, Korea; tnwl0210@naver.com (S.K.); whitealstlr@naver.com (M.P.); tskim0602@naver.com (T.K.); ksha@kangwon.ac.kr (K.-S.H.); 2 Department of Anesthesiology and Pain Medicine, Hanyang University Hospital, Seoul 04763, Korea; irisjy00@hanmail.net; 3 Departments of Obstetrics and Gynecology, School of Medicine, Kangwon National University, Chuncheon, Gangwon-do 24341, Korea; rapidhwang@kangwon.ac.kr; 4 Departments of Neurobiology, School of Medicine, Kangwon National University, Chuncheon, Gangwon-do 24341, Korea; mhwon@kangwon.ac.kr; 5 Department of Life Sciences, College of Natural Sciences, Kangwon National University, Chuncheon, Gangwon-do 24341, Korea; ryoosw08@kangwon.ac.kr; 6 Department of Biochemistry, College of Science and Biotechnology, Yonsei University, Seoul 03722, Korea; ygkwon@yonsei.ac.kr

**Keywords:** preeclampsia, miRNA, diagnostic biomarker, vascular dysfunction

## Abstract

Preeclampsia (PE) is a pregnancy-specific disorder associated with hypertension and proteinuria. Since there is no proven method to treat PE, early prediction and accurate diagnosis are essential for appropriate management of the disease. Thus, reliable biomarkers for diagnosing PE need to be identified and evaluated. We analyzed serum-soluble factors and miRNAs in 92 patients with PE and an equal number of healthy controls to identify new useful biomarkers for PE. Serum miR-31-5p, miR-155-5p, and miR-214-3p levels were significantly elevated in these patients and highly correlated with clinical symptoms of hypertension and proteinuria, whereas the miR-1290-3p level was decreased. The increased miRNAs were upregulated in an NF-κB-dependent manner and suppressed endothelial nitric oxide synthase (eNOS) and placental growth factor (PlGF) expression. The level of each miRNA had greater than 90% diagnostic accuracy, which was further increased by analyzing its ratio relative to that of miR-1290-3p. Taken together, the ratios of miR-31-5p, miR-155-5p, or miR-214-3p to miR-1290-3p may serve as reliable diagnostic or prognostic tools for PE, thereby providing evidence that these miRNAs are promising mechanism-based targets for designing therapeutic and preventive strategies for the clinical management of PE.

## 1. Introduction

Preeclampsia (PE), a pregnancy-specific disease associated with hypertension, proteinuria, thrombocytopenia, or renal insufficiency, is a common disorder affecting 5% to 7% of all pregnancies [1]. It is a major cause of maternal mortality (10–15%) worldwide and accounts for a substantial proportion of low-birth-weight neonates and iatrogenic preterm delivery [2,3].

The incidence of PE has persistently increased in the past decade due to an increase in the prevalence of predisposing factors, such as maternal age, chronic hypertension, diabetes, renal disease, and obesity [3]. Although the pathogenesis of PE is still enigmatic, increasing evidence reveals that inadequate placentation due to the deficient trophoblastic invasion of the uterine spiral arteries elicits placental hypoxia, immune activation and proinflammatory cytokine production, decreased levels of angiogenic factors, and increased levels of antiangiogenic factors, resulting in the acceleration of the clinical symptoms of PE [4]. Much research into the pathogenesis and mechanism of PE has been conducted; however, its occurrence, development, and morbidity have remained unchanged over the past decade. Therefore, a precise diagnosis using accurate biomarkers associated with pathogenesis is needed to enable the best possible management and treatment of PE.

PE has been clinically diagnosed in pregnant women with elevated blood pressure (≥140/90 mmHg) and urinary protein excretion (>300 mg/24 h) after 20 weeks of gestation, but these are both secondary features of a primary placental problem and subject to measurement error and poor test accuracy [5]. Therefore, the identification of accurate biomarkers to enable the prognosis of preeclamptic complications could substantially contribute to management strategies, with the aim of minimizing adverse maternal and fetal outcomes. A growing body of studies has demonstrated that PE is highly associated with increased levels of antiangiogenic factors, such as soluble fms-like tyrosine kinase 1 (sFlt-1) and soluble endoglin (sEng), and decreased levels of angiogenic factors, including placental growth factor (PlGF) [5,6,7,8]. Based on this finding, a high sFlt-1/PlGF ratio has been indicated shortly before the onset of PE [9,10]. Therefore, these circulating factors have been recommended as diagnostic and prognostic biomarkers of PE. However, this antiangiogenic state does not always lead to clinical syndromes such as hypertension and proteinuria in patients with PE, although the reasons for this are still unclear [11,12]. Therefore, new specific biomarkers that can be directly linked to the pathogenesis of clinical features, diagnosis, and therapeutic strategies for PE still need to be discovered.

Major discoveries and rapid progress in miRNA research during the past few years have highlighted that the circulating levels of some specific miRNAs are elevated in many types of human diseases and might have great potential in the diagnosis and therapy of many diseases, including PE, in the near future [13,14,15,16]. Our recent studies demonstrated that miRNA (miR)-31-5p and miR-155-5p are detected at high levels in sera from patients with PE. These miRNAs elicit vascular dysfunction associated with hypertension and proteinuria through the impairment of the endothelial nitric oxide synthase/nitric oxide (eNOS/NO) and soluble guanylate cyclase/protein kinase G (sGC/PKG) pathways [15,16,17,18,19]. This suggests that PE-derived miRNAs associated with the impairment of vasorelaxation and vascular remodeling can be used as diagnostic and therapeutic biomarkers of PE. However, no diagnostic value analysis has been reported using miRNAs based on pathogenic mechanisms or symptoms of PE.

In this study, we identified several miRNAs, such as miR-31-5p, miR-155-5p, miR-214-3p, and miR-1290-3p, whose levels were significantly altered in sera from patients with PE. Their levels were highly correlated with clinical symptoms of PE and had high diagnostic accuracy and specificity for PE. Based on these data, we clearly defined their possible roles not only as useful diagnostic biomarkers for suspected PE but also in vascular dysfunction and the pathogenesis of the disease.

## 2. Materials and Methods

### 2.1. Subjects

Approval for the use of human subjects was obtained from the Institutional Review Board of Kangwon National University Hospital, South Korea (KNUH-2017-01-010-005), and informed consent was obtained from all the participants. This prospective case-control study included 92 women with PE and 92 normotensive pregnant women selected at random, who were enrolled in the Department of Obstetrics and Gynecology, Kangwon National University Hospital, between February 2017 and September 2019. Women with pre-existing diabetes mellitus, chronic hypertension, elevated liver enzyme levels, twin or multiple pregnancies, or any evidence of previous medical illness were excluded. PE was defined as systolic blood pressure ≥140 mmHg and/or diastolic blood pressure ≥90 mmHg on at least two occasions 4 h apart but no more than 7 days apart, which developed after 20 weeks of gestation in previously normotensive women; proteinuria was defined as ≥300 mg protein in a 24 h urine specimen, as previously described [20]. No women with pre-existing hypertension, with or without superimposed PE, were included in the present study. All experiments were carried out in accordance with the approved guidelines and regulations, in line with the tenets of the Declaration of Helsinki.

### 2.2. Sample Collection

The serum fractions were obtained according to standard protocols from all participants. Briefly, at the time of clinic attendance, maternal venous blood samples were collected into standard Vacutainer tubes (EDTA, Becton-Dickinson, Franklin Lakes, NJ, USA) and processed within 1 h by centrifugation at 2000× *g* for 10 min at 4 °C. The supernatants were quickly removed, aliquoted, and stored immediately at −80 °C. For analysis, serum samples were thawed on ice and centrifuged at 3000× *g* for 5 min, avoiding traces of red blood cells and other cellular debris that could affect miRNA analysis. Placentas were also obtained after full-term normal (spontaneous vaginal) deliveries, and maternal arteries were isolated from the placental bed from the patients as described previously [15].

### 2.3. Cell Culture and Treatment

Human umbilical vein endothelial cells (HUVECs) were obtained from Cell Systems (Kirkland, WA, USA) and grown in complete media, as previously described [16]. Human trophoblast-derived HTR-8/SVneo cells were cultured in RPMI 1640 medium supplemented with 10% fetal bovine serum. The human intestinal epithelial cell line DLD-1 was obtained from the American Type Tissue Collection and grown in Dulbecco modified Eagle’s medium (DMEM) supplemented with 10% fetal calf serum. The cells were transfected with 80 nM of miScript Inhibitor Negative Control (#1027271; Qiagen, Hilden, Germany), miR-31-5p inhibitor (#MIN0000089; Qiagen, Hilden, Germany), miR-155-5p inhibitor (#MIN0000646; Qiagen, Hilden, Germany), miR-214-3p inhibitor (#MIN0000271; Qiagen, Hilden, Germany), or miR-1290-3p inhibitor (#MIN0005880; Qiagen, Hilden, Germany) using Lipofectamine RNAiMAX (#56532; Invitrogen, Carlsbad, CA, USA) as previously described [16]. HUVECs and HTR-8/SVneo cells were stimulated with tumor necrosis factor (TNF)-α (10 ng/mL, #210-AT; R&D Systems, Minneapolis, MN, USA) in the presence or absence of Bay 11-7092 (5 μM, #196870; Calbiochem, San Diego, CA, USA) for 24 h, and DLD-1 cells were treated with a mixture of TNF-α, interleukin (IL)-1β (20 ng/mL, #201-LB; R&D Systems, Minneapolis, MN, USA), and interferon (IFN)-γ (20 ng/mL, #285-IF; R&D Systems, Minneapolis, MN, USA) for 24 h. These treatment conditions were used according to previous results to observe the optimal response of each cell type to the cytokines [15,16,21].

### 2.4. miRNA Isolation and Quantitative Reverse Transcription Polymerase Chain Reaction (qRT-PCR)

RNA was isolated from serum (200 μL) and cultured cells using the miRNeasy Mini Kit (#217184; Qiagen, Hilden, Germany) according to the manufacturer’s protocol and eluted with 14 μL of nuclease-free water. cDNA for analyzing miRNAs was prepared from 1 μg of total miRNA using a miScript II RT kit (#218161; Qiagen, Hilden, Germany). qRT-PCR was performed with a miScript SYBR Green PCR kit (#218073; Qiagen, Hilden, Germany) according to the manufacturer’s instructions. The levels of miRNAs were analyzed by miScript Primer Assay with target miRNA-specific and universal primers. The relative levels of miRNAs were normalized to SNORD-95. In addition, total mRNA was isolated using TRIzol reagent (#15596018, Invitrogen, Carlsbad, CA, USA) from cultured cells and placental tissues. mRNA levels of *eNOS* in placental bed-derived arteries, inducible nitric oxide synthase (*iNOS*) in placental tissue, and glyceraldehyde-3-phosphate dehydrogenase (*GAPDH*) were determined by HiPi Real-Time PCR 2X Master Mix (SYBR Green, Elpis, Daejeon, Korea) with a Rotor-Gene Q real-time PCR cycler (Qiagen) using target-specific primers as previously described [15]. The primers used in this study are as follows: 5′-GTGGCTGTCTGCATGGACCT-3′ (forward) and 5′-CCACGATGGTGACTTTGGCT-3′ (reverse) for *eNOS*, 5′-TTCTTCGCCAGACCAAACTG-3′ (forward) and 5′-GGAAGTAGGTGAGGGCTTGC-3′ (reverse) for *iNOS*, and 5′-GGGGCTCTCCAGAACATCAT-3′ (forward) and 5′-GGTCAGGTCCACCACTGACA-3′ (reverse) for *GAPDH*.

### 2.5. Measurement of sFlt-1, sEng, PlGF, Nitrite and Nitrate, and cGMP

Serum levels of sFlt-1 (#DVR100B), sEng (#DNDG00), PlGF (#DPG00), TNF-α (#DTA00C), and cyclic guanosine monophosphate (cGMP; #KGE003) were determined according to the manufacturer’s protocol using ELISA kits (R&D Systems, Minneapolis, MN, USA). Serum levels of NO production [NOx, nitrite (NO_2_^−^) + nitrate (NO_3_^−^)] were analyzed using a nitrate reductase-based nitric oxide assay kit (#ab65328; Abcam, Cambridge, MA, USA).

### 2.6. Western Blot Analysis

Harvested cells were suspended in RIPA buffer [50 mM Tris-HCl, pH 8.0, 150 mM NaCl, 1% Nonidet P-40, 0.5% deoxycholic acid, 0.1% sodium dodecyl sulphate (SDS)], and incubated on ice for 30 min for complete cell lysis. Cell debris was removed by centrifugation at 12,000× *g* for 15 min. Lysates (40 μg) were separated by SDS-polyacrylamide gel electrophoresis, and target protein levels were determined by Western blot analysis using antibodies for eNOS (#610297; BD Biosciences, Franklin Lakes, NJ, USA) and iNOS (#NB300-605; Novus Biologicals, CO, USA).

### 2.7. Statistical Analysis

Statistical analyses were undertaken using GraphPad Prism version 6.0 (GraphPad Software, San Diego, CA, USA). All clinical samples, except those for Western blotting, were analyzed in triplicate, and data are expressed as mean ± SEM. Statistical significance was determined using Student’s *t*-test between two groups, and the correlation between the two factors was determined by linear regression analysis. Receiver operating characteristic (ROC) curves were used to analyze the diagnostic utility of differentially expressed miRNAs or produced serum factors. The optimal cut-off point for the plasma miRNA expression level was determined by the Youden index (J = sensitivity + specificity − 1). Statistical significance was established at *p* < 0.05.

## 3. Results

### 3.1. Levels of sFlt-1, sEng, and PlGF in Sera from Patients with PE

**Figure 1:**
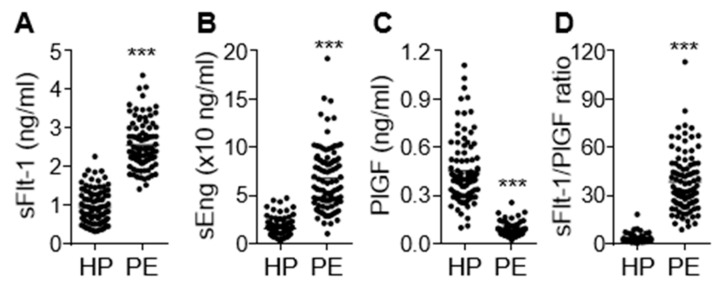
Serum levels of sEng, sFlt-1, and placental growth factor (PlGF) in healthy pregnant women (HP) and preeclampsia (PE) patients (PE). Serum levels of sFlt-1 (**A**), sEng (**B**), and PlGF (**C**) were measured using ELISA (*n* = 92 per group). (**D**) The ratio of sFlt-1 to PlGF. *** *p* < 0.001.

Clinical characteristics of the study participants are shown in Table 1. No significant difference in patient age was noted between healthy pregnant women and preeclamptic patients. However, preeclamptic participants had significantly increased blood pressure (systolic and diastolic, 115.20 ± 0.84 vs. 157.80 ± 1.90 mmHg; 74.89 ± 0.78 vs. 99.85 ± 1.20 mmHg, *p* < 0.001) and proteinuria (1.89 ± 0.19 g/24 h, *p* < 0.001). Moreover, these women delivered low-birth-weight fetuses (3.08 ± 0.06 vs. 2.39 ± 0.07 kg, *p* < 0.001) approximately 1.8 weeks earlier (*p* < 0.001) than the healthy controls. On the basis of the relationship between gestational age and birth weight, the percentage of small for gestational age (SAG) infants (or intrauterine growth retardation; IUGR), appropriate for gestational age (AGA) infants, and large for gestational age (LGA) infants were 29.3%, 68.5%, and 2.2%, respectively, in the women with PE. AGA was defined as a birth weight between 10% and 90% of the average body weight in infants at the same gestational age, while LGA and SGA or IUGR were defined as a birth weight of more than 90% and less than 10% of the average body weight in infants at the same gestational age, respectively [22,23]. Maternal serum concentrations of the antiangiogenic factors sFlt-1 and sEng were significantly higher (*p* < 0.001) in the preeclamptic patients than in the healthy subjects, whereas levels of the angiogenic factor PlGF were decreased in patient sera (*p* < 0.001; Figure 1A–C), consistent with previous reports [6,24]. As expected, the sFlt-1/PlGF ratio, known as a predictive marker of PE, was significantly higher in women with PE than in healthy subjects (*p* < 0.001) and showed a better predictive value than either biomarker alone (Figure 1D). This suggests that the pathological symptoms and analytical characteristics observed in the patients belonged to the typical PE spectrum.

### 3.2. Comparative Levels of eNOS, iNOS, NO, cGMP, and TNF-α in PE Patients

**Figure 2:**
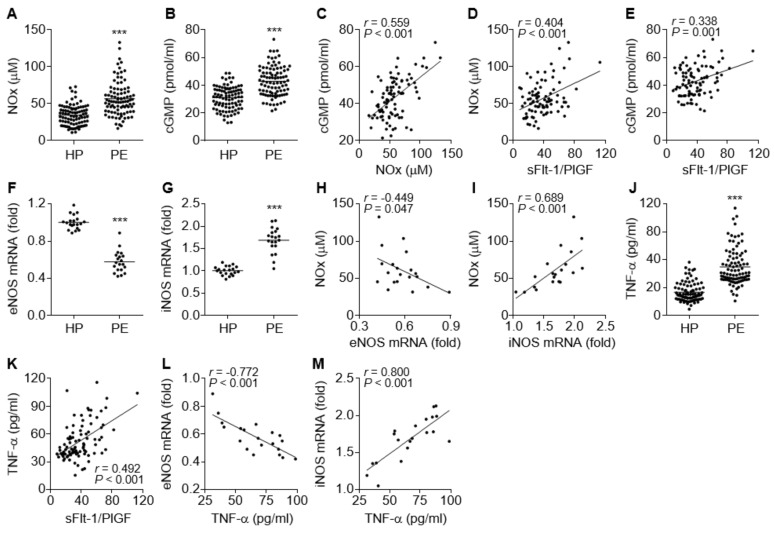
Comparative levels of endothelial nitric oxide synthase (eNOS), inducible nitric oxide synthase (*iNOS*), NOx, cGMP, and TNF-α in healthy pregnant women (HP) and PE patients (PE). (**A**,**B**) Levels of NOx (**A**) and cGMP (**B**) were measured using a nitrate reductase-based nitrated assay kit and an ELISA kit, respectively (*n* = 92). (**C**–**E**) Correlation analysis among the serum levels of NOx, cGMP, and the sFlt-1/PlGF ratio in PE patients (*n* = 92). (**F**,**G**) Expression levels of *eNOS* (**F**) and *iNOS* mRNAs (**G**) were determined in placental tissues from healthy controls and PE patients (*n* = 20). (**H**,**I**) Correlation analysis between NOx level and *eNOS* (**H**) or *iNOS* mRNA levels (**I**) in PE patients (*n* = 20). (**J**) Levels of TNF-α were measured in sera from healthy subjects and PE patients by ELISA (*n* = 92). (**K**–**M**) Correlation analysis between TNF-α levels and the sFlt-1/PlGF ratios (**K**, *n* = 92), as well as between TNF-α levels and *eNOS* (**L**, *n* = 20) or *iNOS* mRNA levels (**M**, *n* = 20), in PE patients. *** *p* < 0.001.

The NO/cGMP axis plays a crucial role in vascular relaxation, and impairment of the axis causes endothelial and vascular dysfunction, which are associated with vascular constriction and glomerular endotheliosis and result in hypertension and proteinuria [25]. We determined the levels of NO and cGMP in the sera of PE patients and control subjects. Unexpectedly, the levels of both molecules were significantly higher in PE patients compared to healthy control subjects (Figure 2A,B). The NOx levels were positively correlated with the cGMP levels (Figure 2C), indicating that the increased cGMP levels are derived by NO-dependent activation of sGC. Both NOx and cGMP levels also positively correlated with the serum sFlt-1/PlGF ratio, a suitable diagnostic marker of PE (Figure 2D,E), indicating that the elevated NO and cGMP levels might be serological features of PE. To investigate which isotype of NOS was responsible for the elevated serum NOx and cGMP levels in the patients, we examined the expression levels of *eNOS* and *iNOS* in umbilical cords and placental tissues immediately after delivery. In contrast to serum NOx and cGMP levels, *eNOS* mRNA levels were significantly decreased in maternal arteries of the placental bed from the PE patients (Figure 2F), whereas *iNOS* expression was significantly increased in their placental tissues (Figure 2G). Furthermore, the elevated NOx levels in the patients were negatively correlated with *eNOS* mRNA levels and positively correlated with *iNOS* mRNA levels (Figure 2H,I), suggesting that the increased NO/cGMP levels in the patients might be derived from increased iNOS expression. Since inflammatory cytokines, including TNF-α, stimulate the iNOS/NO/cGMP pathway [26] and inhibit the eNOS/NO/cGMP pathway [15,16,17,18,19], the relationship between serum levels of TNF-α and *eNOS* or *iNOS* expression was assessed. Our results showed that the serum TNF-α levels were significantly increased in PE patients (Figure 2J) and positively correlated with the sFlt1/PlGF ratio (Figure 2K). More notably, the serum TNF-α levels were inversely correlated with *eNOS* mRNA levels and positively correlated with *iNOS* mRNA levels (Figure 2L,M), suggesting that TNF-α stimulated the iNOS/NO/cGMP pathway but inhibited the eNOS/NO/cGMP axis, probably by activating the transcription factor NF-κB [17,26]. Collectively, these data suggest that PE is likely to be associated with TNF-α-mediated downregulation of eNOS, although total serum NO/cGMP levels are elevated through TNF-α-induced induction of iNOS in preeclamptic patients.

### 3.3. Identification of miRNAs that are Positively and Negatively Regulated in PE Patients

**Figure 3:**
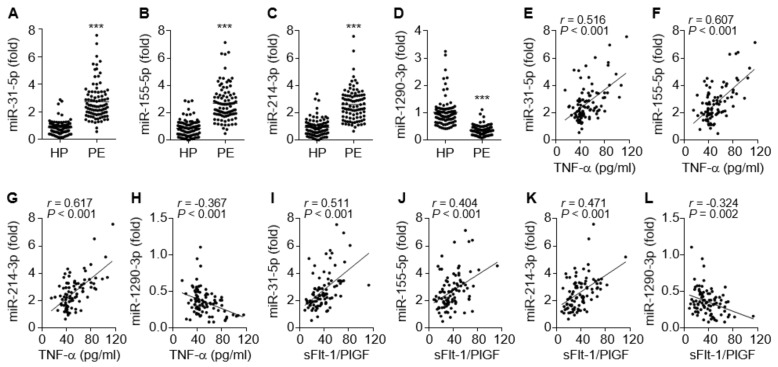
Serum levels of miRNAs in healthy pregnant women (HP) and PE patients (PE), and the correlation between miRNA levels and TNF-α or the sFlt-1/PlGF ratio. (**A**–**D**) Levels of miR-31-5p (**A**) and miR-155-5p (**B**), miR-214-3p (**C**), and miR-1290-3p (**D**) were measured in sera from healthy subjects and PE patients by qRT-PCR. (**E**–**H**) Correlation analysis between TNF-α levels and miRNA levels. (**I**–**L**) Correlation analysis between the sFlt-1/PlGF ratio and the level of each miRNA. *n* = 92 per group. *** *p* < 0.001.

Inflammation-dependent and NF-κB-responsive miR-31-5p and miR-155-5p have been shown to impair endothelial and vascular function via the downregulation of eNOS, sGC, and PKG in the vasculature [16,17,18,19]. Circulating levels of both miRNAs were significantly elevated in PE patients compared to healthy controls (Figure 3A,B), consistent with previous reports [15,16]. Since miR-214 and miR-1290 have been shown to be positively and negatively regulated by hypoxia and estrogen, respectively, which are responsible for the pathogenesis of PE [4,27], we examined the alteration of their expression levels in the sera from patients with PE. The circulating miR-214-3p levels were also increased in the patients (Figure 3C), whereas miR-1290-3p was downregulated (Figure 3D). Notably, miR-31-5p, miR-155-5p, and miR-214-3p levels were highly correlated not only with TNF-α levels but also with the sFlt-1/PlGF ratio of these patients (Figure 3E–G,I–K), whereas miR-1290-3p levels were negatively correlated with TNF-α levels and the sFlt-1/PlGF ratio in the patients (Figure 3H,L). Collectively, these data suggest that increased serum levels of miR-31-5p, miR-155-5p, and miR-214-3p and decreased serum levels of miR-1290-3p are useful biomarkers for the pathogenesis and prognosis of PE.

### 3.4. Correlation between the miRNAs and the NOS/NO/cGMP Axis or PlGF Level in PE Patients

**Figure 4:**
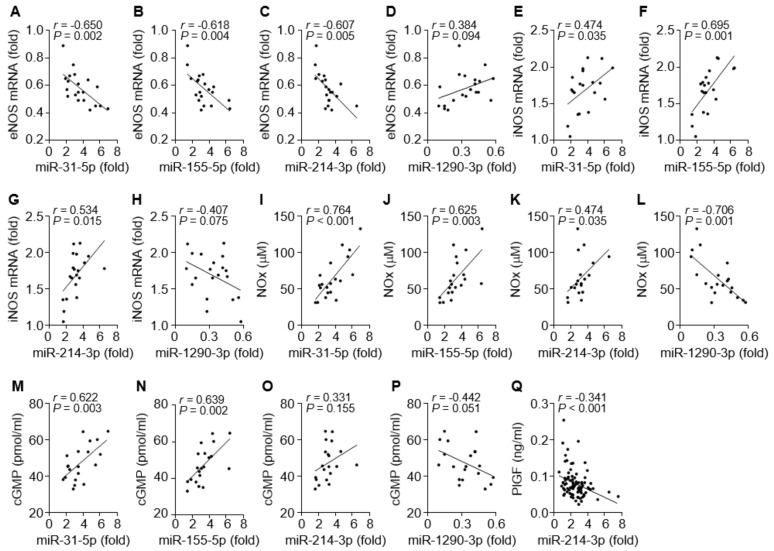
Correlation among *eNOS* mRNA, *iNOS* mRNA, NOx, cGMP, and miRNAs in tissues and sera from PE patients. (**A**–**D**) Correlation analysis between *eNOS* mRNA and miR-31-5p (**A**), miR-155-5p (**B**), miR-214-3p (**C**), or miR-1290-3p level (**D**). (**E**–**H**) Correlation analysis between *iNOS* mRNA and the level of each miRNA. (**I**–**L**) Correlation analysis between NOx level and the level of each miRNA. (**M**–**P**) Correlation analysis between cGMP level and the level of each miRNA. (**Q**) Correlation analysis between PlGF and miR-1290-3p levels. *n* = 20 in **A**–**P** and *n* = 92 in (**Q**).

We next investigated which miRNA is possibly involved in the regulation of the eNOS- or iNOS-dependent NO/cGMP pathway in patients with PE. Circulating levels of miR-31-5p, miR-155-5p, and miR-214-3p were inversely correlated with *eNOS* mRNA levels (Figure 4A–C) and positively associated with the levels of iNOS expression, NOx production, or cGMP synthesis (Figure 4E–G, I–K, and M–O). By contrast, circulating miR-1290-3p levels were partially correlated with the *eNOS* mRNA level and negatively associated with the levels of *iNOS* mRNA, NOx, and cGMP (Figure 4D, H, L, and P). Because miR-214-3p has been shown to target *PlGF* transcripts, we examined the correlation of miR-214-3p and PlGF levels in the sera of PE patients. Serum levels of miR-214-3p were inversely correlated with PlGF levels (Figure 4Q). These data suggest that all four miRNA levels are positively or inversely correlated with the levels of eNOS, iNOS, NO/cGMP, and PlGF in PE patients.

### 3.5. The miRNAs Differentially Regulate iNOS, eNOS, and PlGF Expression

**Figure 5:**
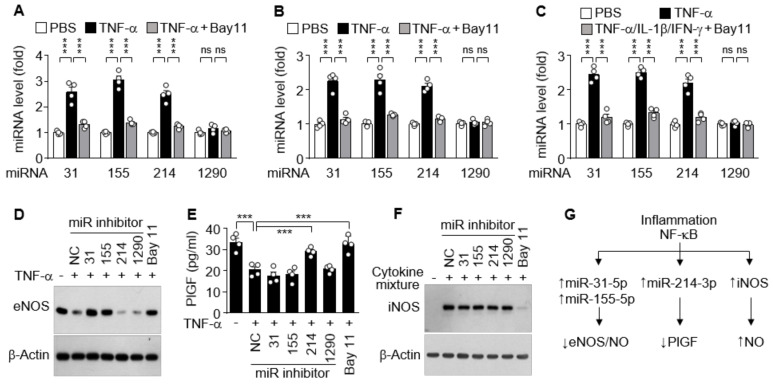
Functional role of cytokine-mediated NF-κB activation in miRNA biogenesis and expression of other genes. (**A**–**C**) HUVECs (**A**), HTR-8/SVneo cells (**B**), and DLD-1 cells (**C**) were treated with TNF-α or TNF-α/IL-1β/IFN-γ in the presence or absence of Bay 11-7082 (Bay11) for 24 h. Levels of the miRNAs were quantified by qRT-PCR (*n* = 4). (**D**–**F**) Cells were transfected with negative control (NC) or miRNA inhibitor, followed by treatment as above. The expression levels of eNOS, PlGF, and iNOS were determined in HUVECs (**D**), HTR-8/SVneo cells (**E**, *n* = 4), and DLD-1 cells (**F**), respectively, by Western blotting and ELISA. (**G**) Schematic diagram depicting the roles of inflammation-associated NF-κB activation in eNOS, PlGF, and iNOS expression. ns, not significant. *** *p* < 0.001.

Since increased cytokine levels in PE patients dysregulate vascular function and trophoblast activity via regulation of the NOS/NO pathway and PlGF production [15,28], we further investigated which miRNA is responsible for the regulation of eNOS, iNOS, and PlGF expression in cell culture systems. Treatment of HUVECs with TNF-α resulted in significant increases in the biogenesis of miR-31-5p, 155-5p, and miR-214-3p, but not of miR-1290-3p, which were abolished by the NF-κB inhibitor Bay 11-7082 (Figure 5A). Similar results were observed in HTR-8/SVneo cells and DLD-1 cells treated with TNF-α alone or in combination with IL-1β and IFN-γ, respectively, in the presence or absence of Bay 11-7082 (Figure 5B,C), suggesting that miR-31-5p, 155-5p, and miR-214-3p, but not miR-1290-3p, are expressed in an NF-κB-dependent manner. As shown in our previous reports [16,17], TNF-α suppressed eNOS expression in HUVECs, and this suppression was rescued by cotreatment with Bay 11-7802 or transfection with an inhibitor of miR-31-5p or miR-155-5p, but not of miR-214-3p and miR-1290-3p (Figure 5D). TNF-α treatment suppressed PlGF production in HTR-8/SVneo cells, which were recovered by treatment with Bay 11-7082 or transfection with miR-214-3p inhibitor, but not with other miRNA inhibitors (Figure 5E). Meanwhile, immune-activated DLD-1 cells induced iNOS expression, which was blocked by treatment with Bay 11-7082, but not by transfection with miRNA inhibitors (Figure 5F). These data indicate that iNOS, eNOS, and PlGF expressions are differentially regulated in the clinical conditions of PE via NF-κB activation, which plays a crucial role in iNOS expression, miR-31-5p/155-5p-mediated eNOS downregulation, and miR-214-3p-mediated PlGF suppression (Figure 5G).

### 3.6. TNF-α and the miRNAs are Associated with Clinical Symptoms of PE

**Figure 6:**
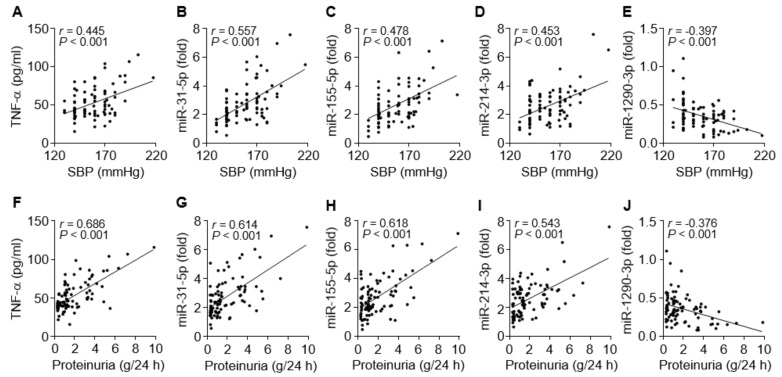
Correlation between miRNA levels and clinical symptoms in PE patients. (**A**–**E**) Correlation analysis between systolic blood pressure (SBP) and TNF-α or the level of each miRNA. (**F**–**J**) Correlation analysis between proteinuria and TNF-α or the level of each miRNA. *n* = 92.

To evaluate the possible links between the circulating miRNA levels and clinical symptoms, such as hypertension and proteinuria, in PE patients, we compared the relationship between them. Systolic blood pressure of the patients was evidently correlated with levels of TNF-α (*r* = 0.445, *p* < 0.001), miR-31-5p (*r* = 0.557, *p* < 0.001), miR-155-5p (*r* = 0.478, *p* < 0.001), and miR-214-3p (*r* = 0.453, *p* < 0.001) and negatively correlated with miR-1290-3p levels (*r* = –0.397, *p* < 0.001; Figure 6A–E). Similar results were observed for correlations between proteinuria levels and TNF-α (*r* = 0.686, *p* < 0.001), miR-31-5p (*r* = 0.614, *p* < 0.001), miR-155-5p (*r* = 0.618, *p* < 0.001), miR-214-3p (*r* = 0.543, *p* < 0.001), and miR-1290-3p (*r* = −0.376, *p* < 0.001; Figure 6F–J). Collectively, these results raise the possibility that increased miR-31-5p, miR-155-5p, and miR-214-3p levels are correlated with clinical characteristics of PE, probably via TNF-α/NF-κB-responsive miR-31-5p/155-5p-mediated downregulation of eNOS and TNF-α/NF-κB-responsive miR-214-3p-mediated suppression of PlGF [29], although miR-1290-3p biogenesis and functions in PE are unknown.

### 3.7. The miRNAs are Useful Serum Factors as Diagnostic Biomarkers in PE

**Figure 7:**
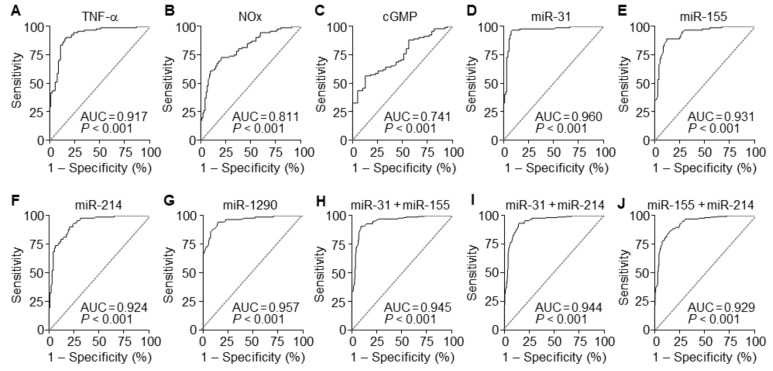
Diagnostic values of soluble factors and miRNAs. (**A**–**D**) ROC curve analysis was performed to evaluate the diagnostic power of TNF-α (**A**), NOx (**B**), and cGMP (**C**). (**D**–**G**) ROC curve analyses of miR-31-5p (**D**), miR-155-5p (**E**), miR-214-3p (**F**), and miR-1290-3p (**G**) in the discrimination of PE patients from healthy controls. (**H**–**J**) ROC curve analyses of the combination of miR-31-5p + miR-155-5p (**H**), miR-31-5p + miR-214-3p (**I**), and miR-155-5p + miR-214-3p (**J**). *n* = total 184 participants (92 PE patients and 92 healthy controls) per soluble factor or miRNA.

To evaluate the diagnostic value of circulating TNF-α, NOx, and cGMP for PE, ROC curve analysis was performed. A comparison of PE patients and normal pregnant subjects showed that serum levels of TNF-α, NOx, and cGMP had the area under the ROC curve (AUC) of 0.917 (95% CI 0.876–0.985), 0.811 (95% CI 0.750–0.873), and 0.741 (95% CI 0.655–0.826), with a sensitivity of 90.22%, 72.83%, and 88.04% and specificity of 83.70%, 79.35%, and 43.59%, respectively (Figure 7A–C and Table 2). These results suggest that among these soluble factors, TNF-α has better diagnostic value than others in the sera of suspected patients with PE. TNF-α has been known to elicit endothelial dysfunction associated with hypertension through the downregulation of eNOS by upregulating NF-κB-responsive miR-31-5p and miR-155-5p [15,16]. Thus, we next compared the diagnostic accuracy of the miRNAs between sera of PE patients and healthy controls using ROC curve analyses. Individual miR-31-5p, miR-155-5p, miR-214-3p, and miR-1290-3p had AUC values of 0.960 (95% CI 0.931–0.990), 0.931 (95% CI 0.895–0.967), 0.924 (95% CI 0.887–0.962), and 0.957 (95% CI 0.931–0.984), with a sensitivity of 95.65%, 89.13%, 90.22%, and 94.57% and specificity of 92.39%, 88.04%, 79.35%, and 84.78%, respectively (Figure 7D–G and Table 2). We further analyzed the synergistic diagnostic power by combination analysis among these miRNAs, except miR-1290-3p, since it was downregulated in the patients. Combinations of miR-31-5p and miR-155-5p, miR-31-5p, and miR-214-3p, and miR-155-5p and miR-214-3p yielded AUC values of 0.945 (95% CI 0.922–0.968), 0.944 (95% CI 0.921–0.967), and 0.929 (95% CI 0.903–0.955), with a sensitivity of 90.76%, 93.48%, and 86.96% and specificity of 90.76%, 85.33%, and 84.24%, respectively (Figure 7H–J and Table 2). These results suggest that these individual miRNAs are potential biomarkers with greater than 90% diagnostic accuracy, which was not further increased by their combined analysis.

### 3.8. The Ratios of miR-31-5p, miR-155-5p, and miR-214-3p to miR-1290-3p Improve Diagnostic Accuracy

**Figure 8:**
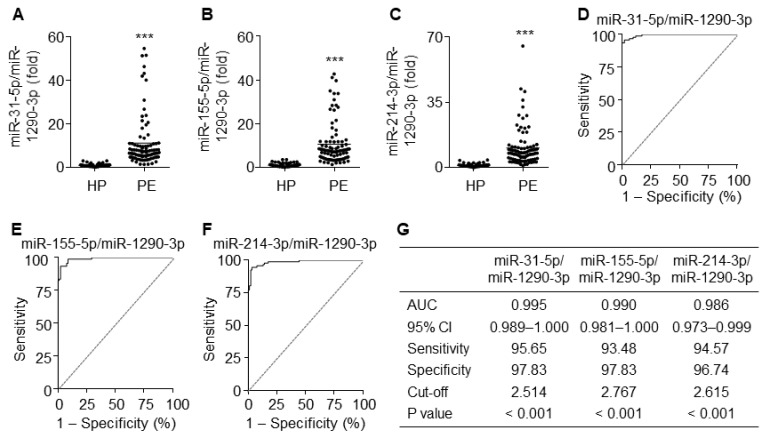
Diagnostic values for the ratios of miR-31-5p, miR-155-5p, and miR-214-3p levels to miR-1290-3p level. (**A**–**C**) Comparative ratios of miR-31-5p (**A**), miR-155-5p (**B**), and miR-214-3p levels (**C**) to miR-1290-3p level in PE patients (PE) and healthy pregnant women (HP). *n* = 92 per group, *** *p* < 0.001. (**D**–**F**) ROC curve analysis of miR-31-5p/miR-1290-3p ratio (**D**), miR-155-5p/miR-1290-3p ratio (**E**), miR-214-3p/miR-1290-3p (**F**) in the discrimination of PE patients from healthy pregnant women. *n* = total 184 participants (92 PE patients and 92 healthy controls) per miRNA. (**G**) Summary of the diagnostic values of the miR-31-5p/miR-1290-3p ratio, miR-155-5p/miR-1290-3p ratio, and miR-214-3p/miR-1290-3p ratio.

Since miR-1290-3p was downregulated in patients with PE, we next analyzed the diagnostic values using the ratios of upregulated miR-31-5p, miR-155-5p, and miR-214-3p to miR-1290-3p. As expected, the ratios of all three miRNAs to miR-1290-3p were significantly higher in PE patients than in healthy pregnant women (Figure 8A–C). ROC curve analysis using the ratios of miR-31-5p, miR-155-5p, and miR-214-3p to miR-1290-3p yielded AUC values of 0.995 (95% CI 0.989–1.000), 0.990 (95% CI 0.981–1.000), and 0.986 (95% CI 0.973–0.999), with a sensitivity of 95.65%, 93.48%, and 94.57% and specificity of 97.83%, 97.83%, and 96.74%, respectively (Figure 8D–G). These results suggest that these ratios have better potential diagnostic power with over 95% accuracy for PE than miRNAs individually or in combination.

## 4. Discussion

It is well-known that placental lesions and uteroplacental vascular insufficiency cause an imbalanced angiogenic state, such as high sFlt1 and sEng levels and low PlGF levels, which are associated with the pathogenesis of PE [6,8]. Accumulating evidence has demonstrated that the sFlt-1/PlGF ratio is significantly increased in maternal blood in the early stage of the clinical development of PE and is considered a diagnostic biomarker [9,10]. This suggests that circulating levels of sFlt-1 and PlGF can be used as pathogenic risk factors and diagnostic and prognostic biomarkers of PE. Unfortunately, pregnant women with a high sFlt-1/PlGF ratio do not always develop hypertension and proteinuria [11,12]. Therefore, new accurate biomarkers directly linked to the clinical symptoms of PE are needed to improve their predictive values.

PE is a pregnancy-specific vascular disease that is also considered an inflammatory disorder caused by the elevation of circulating proinflammatory cytokines in the patients [30,31,32]. We and others have previously demonstrated that serum levels of inflammatory cytokines, including TNF-α, which induces vasoconstriction and hypertension, are significantly elevated in the patients [15,16,17,18,19,33]. Infusion of TNF-α into pregnant rodents and nonhuman primates induces PE-like syndrome [24,34], implying the critical involvement of TNF-α in the pathogenesis of PE. In this study, we also found a potential role of TNF-α in the pathogenesis of PE by confirming a correlation between TNF-α levels and the sFlt-1/PlGF ratio or clinical symptoms. In addition, circulating miR-31-5p, miR-155-5p, and miR-214-3p levels were increased in PE patients and highly correlated with TNF-α levels, the sFlt-1/PlGF ratio, and clinical symptoms. In contrast, miR-1290-3p levels were decreased and inversely correlated with clinical symptoms, although miR-1290-3p expression was not altered by TNF-α and NF-κB activation. Collectively, our data suggest that these miRNAs are promising diagnostic or prognostic biomarkers for PE. However, to utilise the miRNAs as predictive factors of PE, further studies are needed for the prospective analysis of the predictive and correlative values between the circulating mRNA levels before the onset of symptoms and late-onset PE.

The eNOS/NO pathway plays a crucial role in vasorelaxation, angiogenesis, and vascular remodeling. Thus, inhibition or knockdown of eNOS elicits PE-like syndromes, such as hypertension and proteinuria, in rats and mice [25,35,36]. This suggests that impairment of the eNOS/NO/cGMP axis causes endothelial dysfunction, which is directly associated with the pathogenesis of PE. More recent studies showed that elevated TNF-α in PE is responsible for the NF-κB-mediated biogenesis of miR-31-5p and miR-155-5p and subsequent inhibition of the eNOS/NO axis in cultured human endothelial cells, resulting in the impairment of vascular relaxation and trophoblast invasion [15,16]. Similarly, our data demonstrated a strong correlation between TNF-α levels and miR-31-5p or miR-155-5p level and a concomitant decrease in eNOS expression in PE subjects. We also directly demonstrated that TNF-α inhibits eNOS expression in HUVECs by NF-κB-dependent biogenesis of miR-31-5p and miR-155-5p. These findings suggest that preeclamptic hypertension can be caused by the downregulation of the eNOS/NO axis via NF-κB-mediated biogenesis of miR-31-5p and miR-155-5p.

NF-κB-regulated miR-155-5p downregulates the NO-dependent sGC/cGMP/PKG axis in vascular smooth muscle cells (vSMCs) [18,19], leading to additional vasocontraction and hypertension. Unexpectedly, we found that serum NOx and cGMP levels were elevated in PE, although their levels have been shown to be equivocally altered, i.e., decreased, increased, or remained unchanged, in the blood of PE patients [37]. These phenomena might be associated with the differential expression of eNOS and iNOS via the activation of NF-κB in response to inflammatory cytokines. Indeed, NF-κB is significantly activated in the placenta in PE patients [38]. NF-κB has dual functions in pathophysiological NO production, through the transcriptional induction of iNOS in various types of cells, including vSMCs, hepatocytes, and macrophages [39], and the post-translational downregulation of eNOS in endothelial cells via the biogenesis of miR-31-5p and miR-155-5p [16,17]. In general, iNOS is able to synthesize high (micromolar) amounts of NO for many hours or even days, whereas eNOS produces lower (picomolar) and transient levels of NO. Therefore, increased NOx/cGMP levels in PE are thought to be due to the significant induction of iNOS, although eNOS was downregulated. Because NO synthesized by eNOS and iNOS has different biological functions, such as vascular functions and immune regulation, respectively [39], the decreased eNOS/NO pathway is responsible for hypertension and proteinuria in PE, while the elevated iNOS levels stimulate immune activation and tissue damage. Based on all these findings, increased NOx/cGMP levels are due to NF-κB-dependent iNOS induction and are unlikely to be linked to the regulation of vascular function. Thus, NOx and cGMP levels are not better diagnostic biomarkers for PE than miR-31-5p and miR-155-5p levels, which are associated with vascular dysfunction by downregulating eNOS, sGC, and PKG in the endothelial cells and vSMCs of PE patients.

Besides miR-31-5p and miR-155-5p, we also identified that miR-214-3p was significantly elevated in the sera of the patients and upregulated in trophoblasts stimulated with TNF-α. miR-214-3p is located on the opposite strand of an intron of dynamin 3 [40], and its expression is modulated by NF-κB (Figure 5) and hypoxia-inducible factor (HIF)-1α [41]. Given the evidence that inflammation and hypoxia are crucial risk factors for PE [4,32], miR-214-3p is expected to be increased in women with PE. Although miR-214-3p has been shown to be equivocally linked to PE, with microarray data showing its downregulation and qRT-PCR data indicating its upregulation [41], we demonstrated that miR-214-3p was upregulated in an NF-κB-dependent manner in the human trophoblast cell line HTR-8/SVneo treated with TNF-α. Consistent with a previous study [29], our data showed that NF-κB-dependent miR-214-3p biogenesis is responsible for TNF-α-mediated suppression of PlGF production, suggesting that miR-214-3p expression precedes the downregulation of PlGF and is an earlier and better diagnostic biomarker of PE than PlGF. We also found that there is a strong inverse correlation between circulating levels of miR-214-3p and PlGF in PE patients. PlGF is highly expressed in the placenta in all gestational stages and promotes trophoblast invasion into the decidua, as well as spiral artery remodeling. Therefore, increased miR-214-3p levels can cause preeclamptic characteristics via PlGF downregulation and defects in spiral artery remodeling, thereby representing a reliable biomarker for the early diagnosis of PE.

miR-1290-3p is upregulated in tumor stem cells [42] or downregulated during neuronal differentiation [43], suggesting that it somehow participates in cell division and cellular differentiation in the embryo. A recent study showed that miR-1290-3p was highly upregulated in the endometrium on Day 3 of the bovine estrous cycle, followed by rapid downregulation [44], suggesting that its level is regulated in an ovarian hormone-responsive manner and that it plays an important role in endometrial contribution to the implantation and survival of embryos. In fact, miR-1290-3p expression is downregulated in estrogen receptor α-positive breast cancer tumors [45], indicating that estrogens negatively regulate miR-1290-3p. Notably, estrogen concentrations were elevated in PE versus healthy controls [27,46,47]. This suggests that miR-1290-3p is downregulated in PE, consistent with our present findings. Although its expression regulation and target genes are largely unknown in PE, miR-1290-3p can be used as a downregulated biomarker of PE, similar to PlGF.

**Figure 9:**
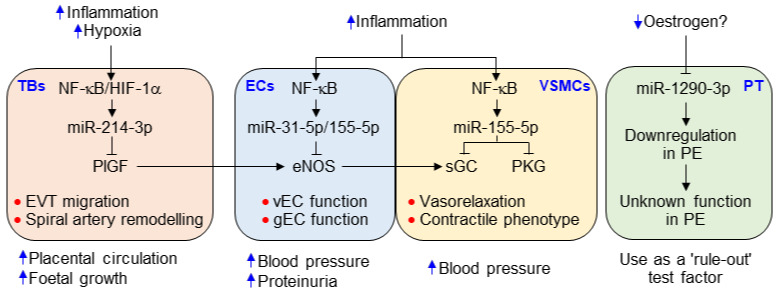
Possible roles of the identified miRNAs in the pathogenesis of PE. Hypoxia, inflammation, and decreased estrogen levels caused by inappropriate implantation. Inflammation and hypoxia increase the biogenesis of miR-31-5p, miR-155-5p, and miR-214-3p in endothelial cells (ECs), vSMCs, and the trophoblast (TB) via activation of NF-κB and HIF-1α. They inhibit the expression of eNOS in ECs [16,17,18], sGC and PKG [19,20] in vSMCs, and PlGF in the TB (Figure 5), resulting in the impairment of vasorelaxation and TB invasion, which cause hypertension, proteinuria, and spiral artery remodeling. Meanwhile, reduced estrogen levels downregulate miR-1290-3p in placental tissue (PT); the function of miR-1290-3p, however, has not been identified in the development of PE. Nevertheless, decreased miR-1290-3p level is considered a “rule-out” test factor, like the reduced PlGF level that is used as a critical factor to discriminate against women with suspected PE from healthy pregnant women.

The pathogenesis of PE occurs as a consequence of interactions among multiple factors, including hypoxia, the immune system, the hormonal system, and oxidative stress [48]. Thus, a single factor is not sufficient to explain the pathogenic mechanism or determine the diagnosis of PE. The strengths of this study include the identification of multiple mechanism-based miRNAs, including inflammation-responsive miR-31-5p and miR-155-5p, and NF-κB/HIF-α-dependent miR-214-3p and miR-1290-3p (Figure 9). Their serum levels were highly correlated with the sFlt-1/PlGF ratio and clinical symptoms and had high diagnostic accuracy as demonstrated by ROC value analysis. However, miR-155-5p has been shown to be elevated in several pathogenic conditions, such as cancer, inflammatory disorders, obesity, and diabetes [49,50], suggesting that miR-155-5p is useful, but not ideal, for the diagnosis for PE. The other miRNAs are considered more specific to PE than miR-155-5p because they are expressed to a limited extent in few other pathological conditions. More notably, all the miRNAs, except miR-1290-3p, the function of which is unknown, are considered risk factors for the development of PE and are thus useful as mechanism-based biomarkers for diagnosing PE. Because miR-1290-3p, considered an inflammation/NF-κB-insensitive and estrogen-responsive biomarker, was downregulated in PE patients, the ratios of miR-31-5p, miR-155-5p, or miR-214-3p to miR-1290-3p may be clinically useful as effective diagnostic determinants for PE, as shown by the diagnostic value of the sFlt1/PlGF ratio. Using the current analytical techniques with qPCR and silver nanocluster DNA probes [51], these miRNAs can be analyzed quickly in a small volume of blood, representing a test that could aid the risk stratification of women with suspected PE.

In conclusion, we identified that the serum levels of several biomarkers were significantly elevated in patients with PE. Among them, NF-κB-responsive miR-31-5p, miR-155-5p, and miR-214-3p could be used as novel mechanism-based diagnostic biomarkers and therapeutic targets for PE (Figure 9). Furthermore, the ratios of those miRNAs to miR-1290-3p, which was reduced in PE, showed greater diagnostic power than that of their individual miRNA, providing evidence that these ratios might be of potential value in the diagnosis and prognosis of women with suspected PE, although the case numbers are still too low to make sufficient differentiation.

## Figures and Tables

**Table 1:** Clinical parameters of normal and preeclamptic pregnancies.

Characteristics	Healthy pregnancy (*n* = 92)	Preeclampsia (*n* = 92)	*p*-Value
Age at pregnancy (year)	31.49 ± 0.50	32.73 ± 0.54	0.0946
Gestational age at delivery (week)	37.52 ± 0.38	35.72 ± 0.28	0.0002
Systolic blood pressure (mmHg)	115.20 ± 0.84	157.80 ± 1.90	<0.0001
Diastolic blood pressure (mmHg)	74.89 ± 0.78	99.85 ± 1.20	<0.0001
Urinary protein (g/24 h)	N/A	1.89 ± 0.19	N/A
Birth weight (kg)	3.08 ± 0.06	2.39 ± 0.07	<0.0001

Values are presented as mean ± SEM. N/A = not available, *n* = 92 per group.

**Table 2:** Diagnostic values of miRNAs and other factors between PE patients and healthy controls.

Serum Factors	AUC	95% CI	Sensitivity	Specificity	Cut-off	*p*-Value
TNF-α	0.917	0.876–0.958	90.22	83.70	34.89	<0.001
NOx	0.811	0.750–0.873	72.83	79.35	44.59	<0.001
cGMP	0.741	0.655–0.826	88.04	43.59	31.77	<0.001
miR-31-5p	0.960	0.931–0.990	95.65	92.39	1.275	<0.001
miR-155-5p	0.931	0.895–0.967	89.13	88.04	1.365	<0.001
miR-214-3p	0.924	0.887–0.962	90.22	79.35	1.250	<0.001
miR-1290-3p	0.957	0.931–0.984	94.57	84.78	0.595	<0.001
miR-31-5p+miR-155-5p	0.945	0.922–0.968	90.76	90.76	1.365	<0.001
miR-31-5p+miR-214-3p	0.944	0.921–0.967	93.48	85.33	1.265	<0.001
miR-214-3p+miR-155-5p	0.929	0.903–0.955	86.96	84.24	1.355	<0.001
